# Adaptive designs in critical care trials: a simulation study

**DOI:** 10.1186/s12874-023-02049-6

**Published:** 2023-10-18

**Authors:** W. Li, V. Cornelius, S. Finfer, B. Venkatesh, L. Billot

**Affiliations:** 1 MRC Biostatistics Unit, East Forvie Building, University of Cambridge, Cambridge, CB2 0QY UK; 2 MRC Clinical Trials Unit at UCL, Institute of Clinical Trials and Methodology, 90 High Holborn, 2nd Floor, London, WC1V 6LJ UK; 3 Imperial Clinical Trials Unit, School of Public Health, Imperial College London, Stadium House, 68 Woodlane, London, W12 7RH UK; 4 The George Institute for Global Health, 1 King Street, Newtown, NSW 2042 Australia; 5 Faculty of Medicine, University of New South Wales, Sydney, NSW 2052 Australia; 6 Faculty of Medicine, Imperial College London, South Kensington Campus, London, SW7 2AZ UK

**Keywords:** Adaptive design, Critical care, Interim monitoring, Interim analysis, Group sequential design, Bayesian, Randomised controlled trial, Statistical simulation

## Abstract

**Background:**

Adaptive clinical trials are growing in popularity as they are more flexible, efficient and ethical than traditional fixed designs. However, notwithstanding their increased use in assessing treatments for COVID-19, their use in critical care trials remains limited. A better understanding of the relative benefits of various adaptive designs may increase their use and interpretation.

**Methods:**

Using two large critical care trials (ADRENAL. ClinicalTrials.gov number, NCT01448109. Updated 12-12-2017; NICE-SUGAR. ClinicalTrials.gov number, NCT00220987. Updated 01-29-2009), we assessed the performance of three frequentist and two bayesian adaptive approaches. We retrospectively re-analysed the trials with one, two, four, and nine equally spaced interims. Using the original hypotheses, we conducted 10,000 simulations to derive error rates, probabilities of making an early correct and incorrect decision, expected sample size and treatment effect estimates under the null scenario (no treatment effect) and alternative scenario (a positive treatment effect). We used a logistic regression model with 90-day mortality as the outcome and the treatment arm as the covariate. The null hypothesis was tested using a two-sided significance level (α) at 0.05.

**Results:**

Across all approaches, increasing the number of interims led to a decreased expected sample size. Under the null scenario, group sequential approaches provided good control of the type-I error rate; however, the type I error rate inflation was an issue for the Bayesian approaches. The Bayesian Predictive Probability and O’Brien-Fleming approaches showed the highest probability of correctly stopping the trials (around 95%). Under the alternative scenario, the Bayesian approaches showed the highest overall probability of correctly stopping the ADRENAL trial for efficacy (around 91%), whereas the Haybittle-Peto approach achieved the greatest power for the NICE-SUGAR trial. Treatment effect estimates became increasingly underestimated as the number of interims increased.

**Conclusions:**

This study confirms the right adaptive design can reach the same conclusion as a fixed design with a much-reduced sample size. The efficiency gain associated with an increased number of interims is highly relevant to late-phase critical care trials with large sample sizes and short follow-up times. Systematically exploring adaptive methods at the trial design stage will aid the choice of the most appropriate method.

**Supplementary Information:**

The online version contains supplementary material available at 10.1186/s12874-023-02049-6.

## Background

Randomised controlled trials (RCTs) play a pivotal role in identifying effective and harmful treatments. In critical care trials, mortality is frequently the event of primary interest and, as a result, trials require a very large number of participants to detect relatively small between-arm treatment effects [1, 2]. Recent work by Granholm et. al provided an overview of past critical care RCTs and discussed current major challenges and recent improvements associated with those trials [3]. A traditional parallel group RCT is designed to recruit a fixed number of participants and, apart from interim analyses focused mainly on safety, will continue until the predefined number of participants have been recruited with the sample size unaffected by evidence that accumulates during the trial. While this design minimises the likelihood of stopping early due to an imbalance in outcomes arising from the play of chance, it is inefficient given late-phase critical care RCTs often taking many years to compete recruitment.

Unlike traditional RCTs that follow a design-conduct-analysis sequence with limited interim analysis, adaptive trials perform pre-planned interim reviews of the data during the conduct of the trial and may adapt the trial design based on those accumulated data [4]. An adaptive design is defined as *‘a clinical trial design which allows for prospectively planned modifications based on accumulating data through the trial’* [5]. Many types of modifications are possible and an ongoing trial can be adapted by recalculating the total sample size, adding or dropping treatments or doses, stopping early for evidence of efficacy or lack of efficacy, altering the treatment allocation ratio, and recruiting only subtypes of patients who appear most likely to benefit from the trial intervention [6].

The ability to be highly flexible while preserving validity and integrity is one of the main advantages of adaptive designs [7]. This flexibility can be attractive to stakeholders’ such as funding agencies and trial sponsors [5]. Furthermore, adaptive designs can have greater statistical efficiency and may estimate the treatment effect more precisely than traditional fixed designs [7]. Importantly, adaptive trials can also be stopped earlier based on interim analyses and may thus be more efficient and ethical as further participants are not recruited to a trial that has already generated sufficient evidence to answer the question posed [6].

Adaptive designs are increasingly popular and a recent review showed that their widespread application to critical care trials has the potential to improve the cost–benefit ratio of clinical trials among critically ill patients, with some increased in complexity in statistical preparation [1, 5]. While a number of previous critical care trials including Centocor: HA-1A Efficacy in Septic Shock (CHESS) [8], Adjunctive Corticosteriod Treatment in Critically Ill Patients with Septic Shock (ADRENAL) [9] and Normoglyceamia in Intensive Care Evaluation-Survival Using Glucose Algorithm Regulation (NICE-SUGAR) [10] have used group sequential designs, they included a limited number of interim analyses with conservative efficacy boundaries and no futility monitoring thus limiting the room for ‘true adaptation’. Other adaptive approaches including unblinded sample size re-estimation [11] and a seamless phase II/III design [12] have also been used. More recently, a number of trials including Randomized, Embedded, Multifactorial Adaptive Platform Trial for Community-Acquired Pneumonia (REMAP-CAP), AGILE and Australasian Covid-19 Trial (ASCOT) have used Bayesian adaptive group-sequential designs motivated by the need to identify COVID-19 treatments as rapidly as possible [13–15].

Despite these recent examples and the increasing use of more advanced adaptive designs, the relative performance of common statistical methods and the effect of the number of interims remains unclear to many triallists. We therefore aimed to examine the impact of various adaptive strategies via a simulation study based on data from two large critical care trials.

## Methods

### Motivating examples

We used two well-known and influential critical care trials as a basis for the simulation work. Both were large scale, multi-centre, parallel group RCTs with 90-day all-cause mortality as the primary outcome. The ADRENAL trial [9] compared the use of intravenous hydrocortisone to placebo for ventilated critically ill patients with septic shock, and the NICE-SUGAR trial [10] investigated the effect of intensive glucose control (blood glucose target 4.5–6.0 mmol/L) compared to conventional glucose control (target less than 10 mmol/L) for adult patients expected to receive treatment in medical-surgical ICUs on more than two consecutive days. The ADRENAL trial recruited 3800 participants and the NICE-SUGAR recruited 6104, the primary outcome was available for 3658 and 6022 participants respectively.

Both trials performed two pre-specified interim analyses to examine early termination for efficacy using conservative Haybittle-Peto group-sequential stopping boundaries with no futility boundaries. Neither trial was stopped prior to completing originally-planned recruitment. At the final analysis, statistically significant and non-significant differences in 90-day mortality were observed for NICE-SUGAR and ADRENAL respectively.

### Group-sequential approaches

Group-sequential designs involve the conduct of interim analyses using pre-specified decision rules for early termination for *efficacy*, or lack of efficacy, often called *futility* [4]. They are the most commonly used interim monitoring approaches due to their relatively high level of understanding by statistical methodologists and clinicians [16], and a long history of practical use [17]. Group-sequential designs require that boundaries for efficacy and futility are prespecified. These boundaries are typically calculated using α- and β-spending functions, where α and β respectively represents the chance of making a type I error and type II error respectively. Spending functions provide a flexible approach that maintains the expected overall type I and type II error rate over the course of the study. One advantage of spending functions is that there is no strict need to pre-specify the number or timing of the interim looks [17]; however, in practice, one would typically expect to indicate the anticipated number of interim analyses at the trial onset.

In this study we compare three group-sequential designs: Haybittle-Peto (HP) [18, 19], O’Brien-Fleming (OBF) [20] and Hwang-Shih-DeCani (HSD) [21]. While the HP design uses constant boundaries, OBF and HSD boundaries are defined by α-spending function that depend on the information rate, defined as the fraction of information accumulated at the time of the interim analysis (i.e. the proportion of the total enrolled sample size) [22] (see Table 1). Compared with common spending functions, HSD uses one additional parameter γ to flexibly set the upper bound (α) spending and lower bound (β) spending. We set the parameters for both upper and lower bound to γ = -4 after examining the spending function to balance the stopping boundaries, making them more conservative than HP in early efficacy monitoring, but less conservative than OBF in both efficacy and futility monitoring. OBF and HSD examine both efficacy and futility stopping by combining α-spending function with β-spending function. HP only considers efficacy monitoring with a fixed critical value of 3 regardless of the number of interims, except the final interim look with the value close to 1.96 (non-group-sequential critical value) [17].

**Table 1:** Spending functions

Spending function	Function forms
O’Brien-Fleming (OBF)	$\mathrm{\alpha }(\mathrm{t})=2-2\upphi (\frac{{\mathrm{Z}}_{1-\frac{\mathrm{\alpha }}{2}}}{\sqrt{\mathrm{t}}}) (\mathrm{t}\ne 0)$
	$$\mathrm{\alpha }(\mathrm{t})= 0 (\mathrm{t }=0)$$
Hwang-Shih-DeCani (HSD)	$\mathrm{\alpha }(\mathrm{t})=\mathrm{\alpha }(\frac{1-{\mathrm{e}}^{-\mathrm{\gamma t}}}{1-{\mathrm{e}}^{-\upgamma }}) (\upgamma \ne 0)$
	$$\mathrm{\alpha }(\mathrm{t})=\mathrm{\alpha t }(\upgamma =0)$$

t represents the fraction of information accumulated at the time of the interim analysis (t = 0 at the start of the trial and t = 1 at the time of the final analysis) which represents the proportion of the total sample size; α(t) represents the significance level at t; Z_1-α/2_ represents the Z-value at the significance level of (1-α/2); ϕ represents the function that converts the Z-value to the corresponding significance level

**Fig. 1:**
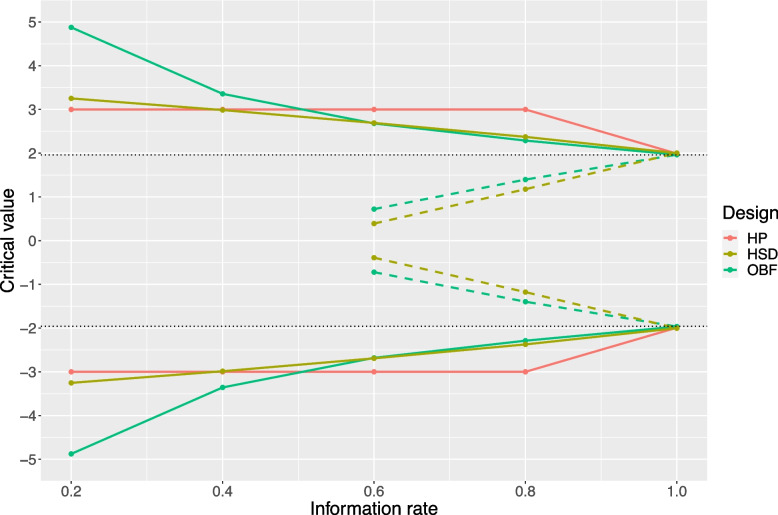
Critical value boundaries of three approaches with four interim looks and one final look. The solid lines represent the efficacy boundaries and the dashed lines represent the futility boundaries for the three group-sequential designs (red: Haybittle-Peto, brown: Hwang-Shih-DeCani, green: O’Brien-Fleming). Orange dashed lines are not shown here due to the absence of futility boundaries for the Haybittle-Peto design

Figure 1 is an example showing the boundaries of the critical values when conducting four equally spaced interim analyses and one final analysis using all three designs. In this study, we employed binding boundaries to strictly stop the trial whenever the efficacy or futility criteria are met. This maintains the validity of the type I and type II error rates associated with the boundaries [23]. The overall α is set to a two-sided value of 0.05. The corresponding β-spending functions are calculated by replacing α with β [23, 24] with a value of 0.1 (90% power).

Critical values and the corresponding α-levels were calculated based on the α-spending functions, depending on the proportion of information available at each interim stage. We compared the standardised z-statistic at the k^th^ interim (Z_k_) with the corresponding critical values at each interim analysis to decide if the trial should be stopped for early efficacy (rejecting the null hypothesis of no between-arm difference) or for early futility (on the acceptance that the trial will be unable to reject the null hypothesis) [17].

###  Bayesian adaptive designs

Bayesian designs are another family of adaptive methods that can be used for interim monitoring. We examined both posterior probabilities (PostP) and predictive probabilities (PredP) as the metrics to make decisions for early stopping [25, 26]. We constructed a Bayesian logistic regression model with a Bernoulli distribution that ${\mathrm{Y}}_{\mathrm{j}}|\mathrm{i }\sim \mathrm{ Bernoulli}({\mathrm{p}}_{\mathrm{i}})$ to model the primary outcome, where ${\mathrm{Y}}_{\mathrm{j}}$ denotes the primary endpoint for patient j and ${\mathrm{p}}_{\mathrm{i}}$ denotes the probability distribution for the primary outcome rate for arm i. In our simulation study, we chose to examine performance for a common case scenario of no prior information and used a vague prior combined with simulated trial data for both arms to obtain the posterior knowledge on the parameters of interest (${\upbeta }_{1}$) within the analysis model: $\mathrm{logit}\left({\mathrm{p}}_{\mathrm{i}}\right)= {\upbeta }_{0}+ {\upbeta }_{1}{\mathrm{x}}_{\mathrm{i}}$, where $\mathrm{logit}({\mathrm{p}}_{\mathrm{i}})$ is the log-odds of having the primary outcome in arm i and x_i_ is a binary treatment assignment indicator; x_i_ = 1 if patient j is allocated to the experimental arm and x_i_ = 0 otherwise (control). The effect of the intervention is estimated as the odds ratio (OR) obtained as exp(${\upbeta }_{1}$). The prior density of $\mathrm{logit}\left({\mathrm{p}}_{\mathrm{i}}\right)$ is assumed to be normally distributed with a mean of 0 and a standard deviation of 4, implying that all response rates between 0 and 100% have a roughly equal probability. [27]. The posterior density of the logarithm of the intervention effect is obtained via Markov Chain Monte Carlo (MCMC) simulation with individual parameters updated by Gibbs sampling, using only the endpoint data available at the time of the update.

We set efficacy threshold to a probability of 99% i.e. by stopping for early efficacy if the probability of the two arms being different (OR > 1 or OR < 1) was greater than 99%. For futility, we used thresholds of 0.85 and 1/0.85 around the OR and stopped if the probability of exceeding both thresholds dropped below 10% (i.e. Pr(OR < 0.85) < 10% and Pr(OR > 1/0.85) < 10%). These thresholds were informed by previous Bayesian adaptive designs including REMAP-CAP [28] which used the same 99% threshold for efficacy and boundaries of 1/1.2 and 1.2 around the OR to declare futility.

### Re-analysis of ADRENAL and NICE-SUGAR

Using actual data collected during the ADRENAL and NICE-SUGAR trials, we retrospectively re-analysed both trials using the three group-sequential approaches and two bayesian approaches. This was done using one, two, four and nine equally spaced interim analyses.

### Simulation study

#### Overview

Our simulation study compared the performance of the five adaptive designs with one, two, four, and nine equally spaced interim analyses.

To ensure realistic scenarios, we followed the original designs for the ADRENAL and NICE-SUGAR trials. We used the same sample size for each trial and the hypothesised treatment effect used by each trial as stated in the original trial protocols. We used the terms *null/alternative scenario* and *null/alternative hypothesis* interchangeably in this simulation study.

We performed the simulation study in R 4.1.0 [29] with the use of rpact 3.3.0 [30] and JAGS 4.3.0 (mcmc-jags.sourceforge.io/). We reported the operating characteristics for each scenario, including the type I error rate, power and sample size to evaluate the performance of each adaptive method.

#### Analysis model and outcome

Interim and final analyses were conducted using an unadjusted logistic regression model with 90-day mortality as the outcome and the randomised arm as the covariate. The effect of the intervention was estimated as the unadjusted ORs.

The null hypothesis for no between-arm treatment difference was tested using a two-sided α-level at 0.05. The null hypothesis is of no treatment difference (*H*_*0*_: OR = 1) against the two-sided alternative that a treatment difference is present (*H*_*1*_: OR > 1 or OR < 1), where OR < 1 is deemed beneficial to the experimental intervention.

#### Data-generating mechanisms

To generate the simulated datasets, we assumed a fixed sample size of 3658 and 6022 unique patients as in the ADRENAL trial and NICE-SUGAR trial, respectively. Interim analyses were assumed to be equally spaced with outcome data available immediately. We also made the assumption that recruitment was paused while an interim analysis was being conducted. We used an equal randomisation ratio (1:1) to ensure balance in the number of patients assigned to either treatment arm during all analyses.

We performed simulations under both the null and alternative hypotheses based on the initial assumptions made for both trials. Under the null scenario, the 90-day mortality rate in both arms was assumed to be 33% for the ADRENAL trial and 30% for the NICE-SUGAR trial. Under the alternative scenario, we assumed an absolute reduction of 5% for the ADRENAL trial (i.e. from 33% down to 28%) and 3.8% for the NICE-SUGAR trial (i.e. from 30% down to 26.2%).

#### Performance measures

The performance of the different adaptive designs was evaluated by deriving the following trial operating characteristics: the type I error rate under the null scenario; the power under the alternative scenario; the probabilities of making an early correct and incorrect decision; the expected sample size. While not the primary focus of the study, we also explored the bias of estimated treatment effect.

The probability of making an early correct decision (ECD) under the null scenario of no treatment difference was defined as the proportion of simulated trials correctly stopped early for futility during interim analyses. Under the alternative scenario of a true treatment effect, the probability of an early correct decision was calculated as the proportion of simulated trials stopped early for efficacy at the time of an interim analysis. Conversely, the probability of making an early incorrect decision (EID) was defined as the proportion of trials stopped for efficacy during all interim analyses under the null scenario or stopped early for futility under the alternative scenario (Table 2). The expected sample size was calculated as the mean sample size of all the simulated trials (i.e. trials that terminated early for efficacy or futility and trials that reached the end of the study). We calculated the probability of stopping for efficacy P(E) and the probability of stopping for futility P(F) at each stage.

**Table 2:** The probability of making an early correct and early incorrect decision under both scenarios

	**Null scenario (no difference)**	**Alternative scenario (true treatment difference)**
Early correct decision (ECD)	Stopped early for *futility* during interim analyses	Stopped early for *efficacy* during interim analyses
Early incorrect decision (EID)	Stopped early for *efficacy* during interim analyses	Stopped early for *futility* during interim analyses

#### Number of repetitions

To ensure sufficient precision and average out random fluctuation [31], we ran 10,000 simulations under the null and alternative scenarios for both studies.

## Results

### Retrospective analysis of ADRENAL trial and NICE-SUGAR trial

**Fig. 2:**
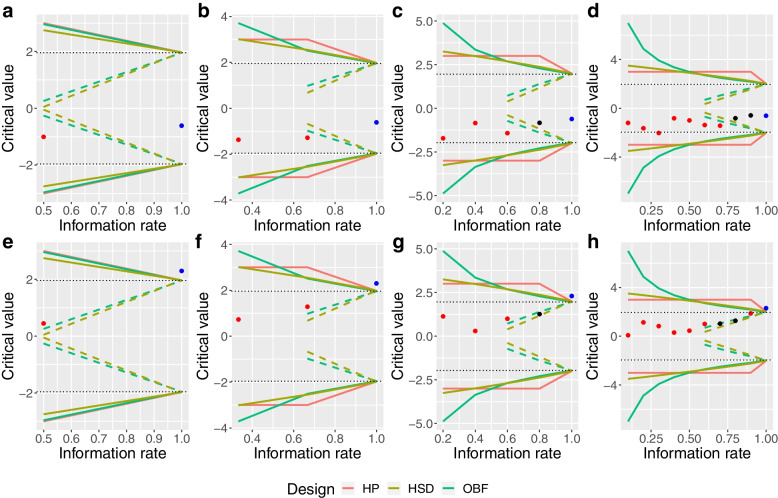
Retrospective analysis using group-sequential designs. Panels **A**,** B**,** C** and** D** show the critical values, represented as dots (red: no boundary crossing, black: boundary crossing, blue: final stage) at each interim or final analysis with stopping boundaries of all group-sequential designs (red: Haybittle-Peto, brown: Hwang-Shih-DeCani, green: O’Brien-Fleming) across different numbers of interim analyses conducted for the ADRENAL trial, respectively. Panels **E**,** F**, **G** and** H** also show the same information for the NICE-SUGAR trial

Our re-analysis results suggested that neither trial would have been stopped early for either efficacy or futility with only one or two interim analyses conducted. However, with four interim analyses, the ADRENAL trial would have been stopped for futility using either the O’Brien-Fleming or Hwang-Shih-DeCani design and the NICE-SUGAR trial would have been stopped for futility using the O’Brien-Fleming design when the information ratereached 80%. When conducting nine interim analyses, NICE-SUGAR would also have been terminated for futility when the information rate reached 70% using the O’Brien-Fleming design (Fig. 2).

### Simulation results

#### Number of interims

**Fig. 3:**
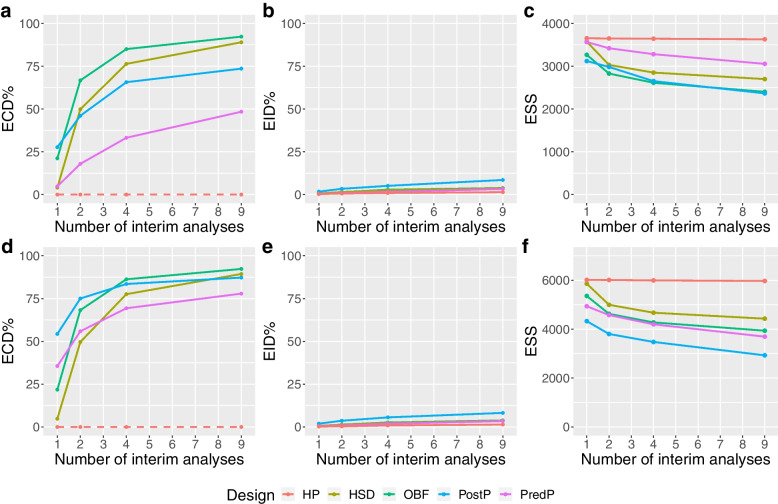
Performance of the designs under the null scenario. Panels **a**,** b** and **c** show the probability of making an early correct decision in percentage, the probability of making an early incorrect decision in percentage and the expected sample size using various designs with different adaptive methods (red: Haybittle-Peto, brown: Hwang-Shih-DeCani, green: O’Brien-Fleming, blue: Posterior probability approach, purple: Predictive probability approach) across different numbers of interim analyses conducted under the null scenario for the ADRENAL trial, respectively. Panels **d**,** e** and** f** show the same metrics for the NICE-SUGAR trial

**Fig. 4:**
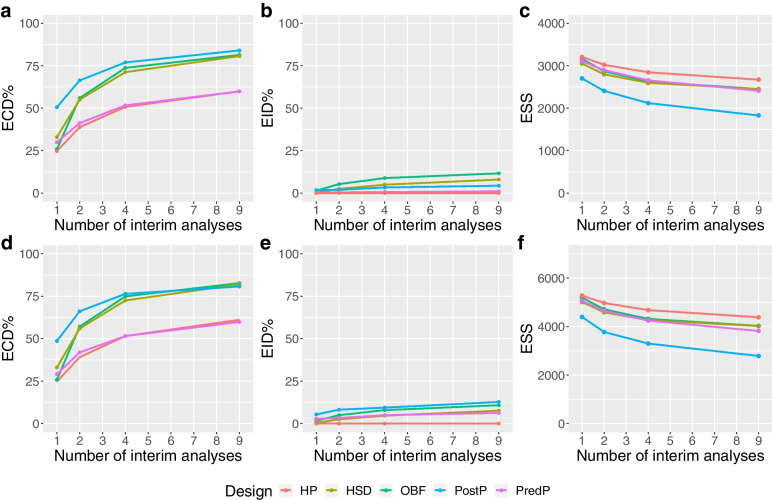
Performance of the designs under the alternative scenario. Panels** a**, **b** and **c** show the probability of making an early correct decision in percentage, the probability of making an early incorrect decision in percentage and the expected sample size using various designs with different adaptive methods (red: Haybittle-Peto, brown: Hwang-Shih-DeCani, green: O’Brien-Fleming, blue: Posterior probability approach, purple: Predictive probability approach) across different numbers of interim analyses conducted under the alternative scenario for the ADRENAL trial, respectively. Panesl **d**, **e** and **f** show the same metrics for the NICE-SUGAR trial

Under both the null and alternative scenarios, the chance of making an early correct decision and the chance of making an early incorrect decision increased as more interim analyses were conducted. This was accompanied by a monotonic decrease in the expected sample size as the number of interims increased (Figs. 3 and 4). Under the null scenario, a single interim analysis led to relative reductions in the expected sample size comprised between 2.4% and 14.7% for the ADRENAL trial and 2.7% and 28.1% for the NICE-SUGAR trial relative to the maximum sample sizes. With nine interims, the reduction in expected sample size was between 16.5% and 35.4% and 26.5% and 51.4%, for ADRENAL and NICE-SUGAR respectively. The HP design resulted in a less than 1% of decrease in the expected sample size under the null scenario due to the absence of futility boundaries. Under the alternative scenario, all group-sequential approaches and Bayesian approaches showed a considerable reduction of about 27% to 53% in the expected sample size for both trials when performing nine interim analyses, compared to a slight decrease of about 12% to 27% with only one interim (See Additional file 1).

##### Null scenario

Under the null hypothesis, one would expect interim analyses to lead to early stopping due to futility (no effect). As the number of interims increased from one to nine, the chance of making an early correct decision (stop for early futility) increased by about 60% for both trials while the chance of making an early incorrect decision (stop for early efficacy) for both trials increased by about 3.5% (Fig. 3). With nine interim analyses, we found that most designs achieved around 75% chance of making an early correct decision for the ADRENAL trial. For the NICE-SUGAR trial, a 75% chance of correctly stopping the trial early for futility (defined as intensive glucose control was not better than conventional control) was reached with only four interim analyses (Fig. 3). Due to the absence of futility boundaries for the Haybittle-Peto design, there was no opportunity to make an early correct decision under the null scenario as indicated by the flat red dashed lines. In addition, we observed an average reduction in the expected sample size of about 30% as the number of interims increases from one to nine. The type I error rate was maintained at 5% across all group-sequential designs regardless of the number of interim analyses (see Additional file 1).

##### Alternative scenario

Under the alternative hypothesis, one would expect interim analyses to lead to early stopping due to efficacy. With nine interim analyses, the chance of making an early correct decision increased by 40% compared to the one with only one interim analysis, while the chance of making an early incorrect decision (stopping for early futility) only increased by about 5 to 8%. There was an approximately 75% chance of making an early correct decision with four interim analyses, which then increased to over 80% when the number of interim analyses increases to nine across most designs for both trials (Fig. 4). Furthermore, as the number of interims increased from one to nine, we observed a maximum reduction in expected sample size for both trials. With nine interim analyses, the expected sample sizes of both trials were reduced to about 50% of the originally planned sample sizes. We also found that most designs achieved a statistical power at 90% across different numbers of interims (see Additional file 1).

#### Choice of design

##### Null scenario

**Fig. 5:**
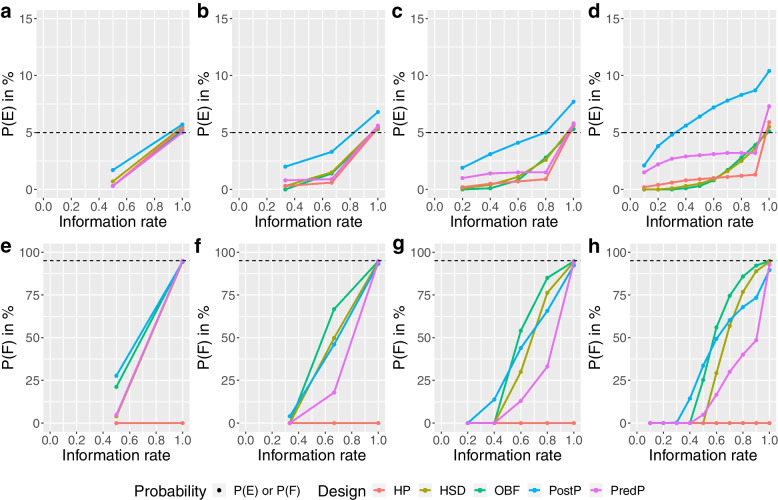
Cumulative probability of stopping for efficacy (P(E)) or futility (P(F)) at each stage under the null scenario for the ADRENAL trial. Panels **a**,** b**,** c** and **d** show the cumulative probability of stopping for efficacy (P(E)) in percentage at each stage and panels **e**,** f**,** g** and **h** show the cumulative probability of stopping for futility (P(F)) in percentage at each stage under the null scenario for the ADRENAL trial using various designs with different adaptive methods (red: Haybittle-Peto, brown: Hwang-Shih-DeCani, green: O’Brien-Fleming, blue: Posterior probability approach, purple: Predictive probability approach). The reference levels at 5% for the P(E) and 95% for the P(F) under the null scenario are shown in dashed lines

All group-sequential designs achieved the desired type I error rate. The PostP design showed an inflated type I error rate with an overall probability of stopping for efficacy (P(E)) well above 5% with more than one interim analysis for both trials. With four or more interims, a type I error rate inflation also occurred using the PredP design (see Fig. 5 and Additional file 1).

**Fig. 6:**
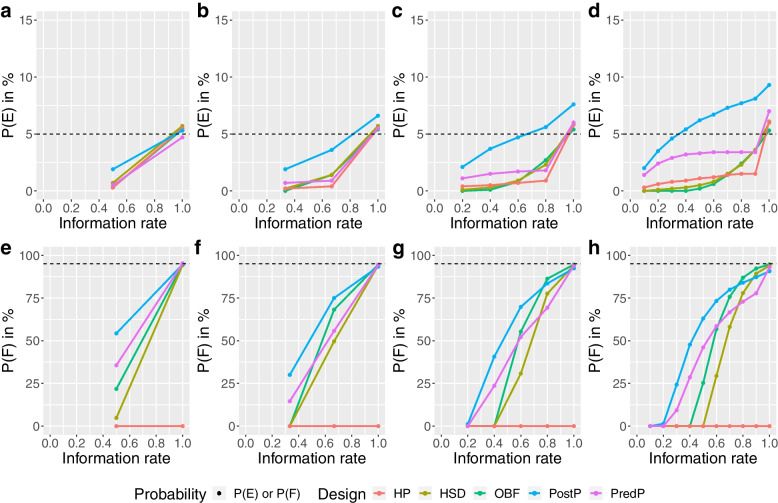
Cumulative probability of stopping for efficacy (P(E)) or futility (P(F)) at each stage under the null scenario for the NICE-SUGAR trial. Panels **a**,** b**,** c** and **d** show the cumulative probability of stopping for efficacy (P(E)) in percentage at each stage and panels **e**,** f**,** g** and **h** show the cumulative probability of stopping for futility (P(F)) in percentage at each stage under the null scenario for the NICE-SUGAR trial using various designs with different adaptive methods (red: Haybittle-Peto, brown: Hwang-Shih-DeCani, green: O’Brien-Fleming, blue: Posterior probability approach, purple: Predictive probability approach). The reference levels at 5% for the P(E) and 95% for the P(F) under the null scenario are shown in dashed lines

The HP design showed the lowest chance of making both an early correct decision and an early incorrect decision and hence had the largest expected sample size. The OBF design showed the highest chance of correctly stopping the ADRENAL trial early for futility and the smallest expected sample size among all designs that achieved a desired type I error rate (Figs. 3 and 5). For the NICE-SUGAR trial, with four or nine interim analyses, the OBF design also showed the highest chance of making an early correct decision and the smallest expected sample size (a reduction of 29%-34% from the maximum sample size) among all designs that achieved a desired type I error rate, with the overall P(E) just above 5% (i.e. overall P(F) just below 95%) (See Fig. 6 and Additional file 1).

For the ADRENAL trial, the PredP design showed a less than 25% chance of making an early correct decision and a less than 1% chance of making an early incorrect decision with no more than two interim analyses conducted. The expected sample size was reduced by only 2.6%-6.5% of the maximum sample size of the ADRENAL trial. However, for the NICE-SUGAR trial, the PredP design showed a relatively high chance of making an early correct decision (55.9%) and also showed the smallest sample size with a 24% reduction with two interim analyses (See Fig. 3 and Additional file 1). With only one interim, the PostP design achieved the highest chance of making an early correct decision and the greatest reduction in the expected sample size, while maintaining the type I error rate (Figs. 3 and 5). For both trials, with one interim, the PredP design achieved the highest overall P(F) at 95% (i.e. overall P(E) at 5%) (Figs. 5 and 6).

##### Alternative scenario

**Fig. 7:**
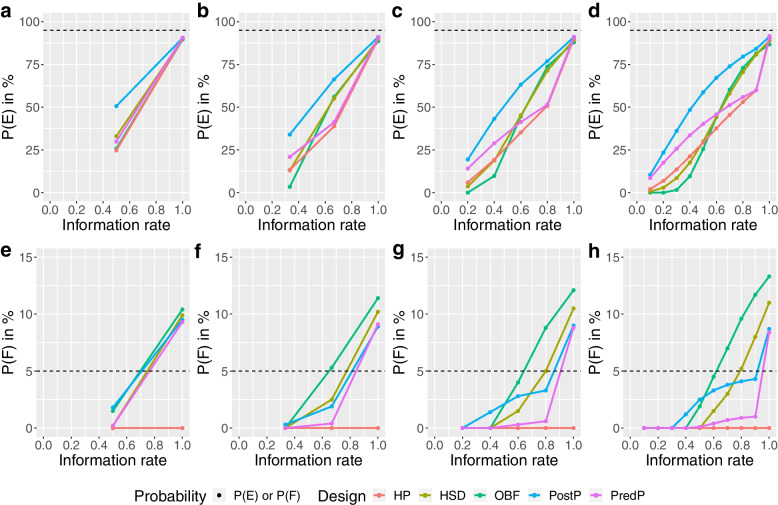
Cumulative probability of stopping for efficacy (P(E)) or futility (P(F)) at each stage under the alternative scenario for the ADRENAL trial. Panels **a**,** b**,** c** and **d** show the cumulative probability of stopping for efficacy (P(E)) in percentage at each stage and panels **e**,** f**,** g** and **h** show the cumulative probability of stopping for futility (P(F)) in percentage at each stage under the alternative scenario for the ADRENAL trial using various designs with different adaptive methods (red: Haybittle-Peto, brown: Hwang-Shih-DeCani, green: O’Brien-Fleming, blue: Posterior probability approach, purple: Predictive probability approach). The reference levels at 95% for the P(E) and 5% for the P(F) under the alternative scenario are shown in dashed lines

**Fig. 8:**
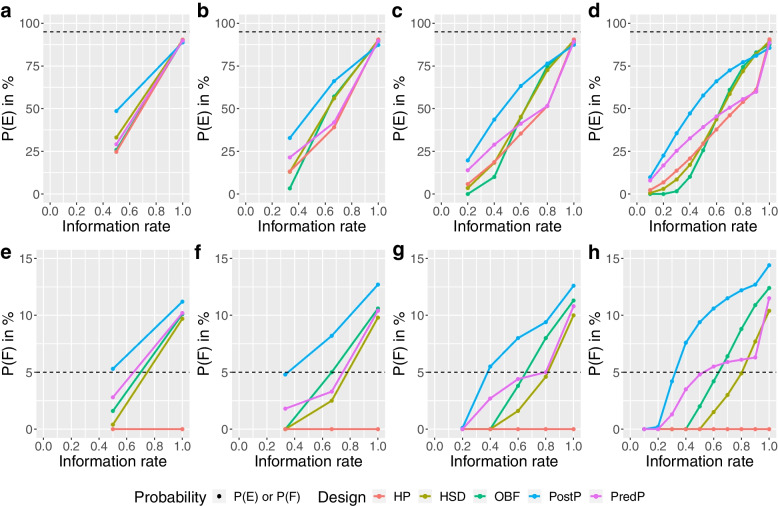
Cumulative probability of stopping for efficacy (P(E)) or futility (P(F)) at each stage under the alternative scenario for the NICE-SUGAR trial. Panels **a**,** b**,** c** and **d** show the cumulative probability of stopping for efficacy (P(E)) in percentage at each stage and panels **e**,** f**,** g** and **h** show the cumulative probability of stopping for futility (P(F)) in percentage at each stage under the alternative scenario for the NICE-SUGAR trial using various designs with different adaptive methods (red: Haybittle-Peto, brown: Hwang-Shih-DeCani, green: O’Brien-Fleming, blue: Posterior probability approach, purple: Predictive probability approach). The reference levels at 95% for the P(E) and 5% for the P(F) under the alternative scenario are shown in dashed lines

As with the null scenario, the HP design reported a relatively low chance of making both an early correct decision and an early incorrect decision, leading to the largest expected sample size across all designs for both trials (Fig. 4). The HP design achieved an overall P(E) around 90.1% for the ADRENAL trial and 90.5% for the NICE-SUGAR trial at different numbers of interims (Figs. 7, 8 and Additional file 1).

The PostP design showed the highest chance of making an early correct decision among all designs with all numbers of interims for the ADRENAL trial and with one, two or four interims for the NICE-SUGAR trial. The PostP design also led to the smallest expected sample size among all designs regardless of the number of interims (Fig. 4). With one, four or nine interims for the ADRENAL trial, the PredP design achieved the highest overall P(E) (i.e. the lowest overall P(F)) (Fig. 7). Despite a relatively low chance of making an early correct decision, the PredP design resulted in a 27.4% reduction in the expected sample size with four interims and 34.1% with nine interims. The HSD and OBF designs achieved similar sample size reductions. For the NICE-SUGAR trial, the HSD design and the OBF design showed the highest chances of making an early correct decision when conducting nine interim analyses (Fig. 4).

### Bias

Under the null scenario, one expects the true OR to be 1.00 (no treatment difference). No bias was apparent from the expected treatment effect calculated as the OR averaged over all simulated trials, compared to the true value suggesting that stopping early for futility under the null scenario does not introduce bias, regardless of the number of interim analyses.

Under the alternative scenario, the true OR was set to 0.79 and 0.83 for the ADRENAL and NICE-SUGAR trials, respectively. Simulations showed that ORs become negatively biased (i.e. further away from 1.0) with the amount of bias increasing as the number of interim analyses increases. The two Bayesian approaches appeared less biased than the group sequential designs when conducting nine interim analyses (average OR 0.74 vs 0.70 for the ADRENAL and average OR 0.80 vs 0.74 for the NICE-SUGAR). With nine interim analyses, the HSD design led to the greatest bias (average OR 0.69 for the ADRENAL and average OR 0.73 for the NICE-SUGAR) (see Additional file 1).

## Discussion

Our analysis shows that applying adaptive methods to critical care trials with and without futility monitoring helps reach trial conclusions with a reduced sample size. The re-analysis of the ADRENAL trial shows that the inclusion of futility boundaries would have led to early stopping for futility with four or nine interim looks using either the O’Brien-Fleming or Hwang-Shih-DeCani design after recruiting 2926 (out of 3658) participants. Thus reaching the same conclusion as the original ADRENAL trial findings with a smaller sample size. The NICE-SUGAR trial might have been stopped for futility with four interim analyses using the O’Brien-Fleming design after recruiting 4817 (out of 6022) participants while early stopping for futility might have occurred with nine interim analyses using the same design after 4215 participants have been recruited. While this represents a substantial saving in sample size, it is important to note that these findings are not consistent with the final conclusion of the original NICE-SUGAR analysis which reported significant harm associated with intensive glucose control. With futility monitoring, the NICE-SUGAR trial would have been stopped early for futility on the basis that intensive glucose control would not be shown to be superior to conventional control. While this reduction in sample size looks attractive, it may have had a negative clinical impact as the absence of definitive evidence of harm would have resulted in continued use of intensive glucose control and in a substantial number of preventable deaths assuming that the originally-reported NICE-SUGAR findings represent the true effect. We used binding futility boundaries in our simulation study; however, it is also possible to use non-binding ones which will provide flexibility to continue the trial even if the boundary is crossed despite a slight increase in sample size.

This re-analysis was followed by an extensive simulation study comparing five interim monitoring approaches under null and alternative scenarios. As previously reported, the results showed that as more interim analyses were conducted, the chance of reaching the correct conclusion before the final analysis stage became higher and the sample size was greatly reduced under both trial settings. This was true under both the null (no treatment effect) and alternative (true treatment effect) scenarios.

Under both scenarios, there was a sharp decrease in the expected sample size as the number of interims increased from one to two and a more gradual and steady decrease as more interim analyses were added. The decrease in the expected sample size was predominantly caused by an increased probability of making an early correct decision. While the impact of the number of interim analysis on the expected sample size is well-known, these findings are of particular relevance to late-phase critical care trials given their large sample size and relatively short follow-up times.

Our simulations confirmed that group-sequential designs provide good control of the type I error rate regardless of the number of interim analyses; however, the two Bayesian approaches failed to maintain the type I error rate when increasing the number of interim analyses. Shi and Yin have proposed novel methods to effectively maintain the overall type I error rate for Bayesian sequential designs [32]. Despite using the same sample sizes as the ones used in the original studies, statistical power was generally well maintained at 90% across all approaches. Notably, the HP design always achieves the highest power regardless of the number of interim analyses while maintaining a fixed sample size due to the conservative stopping boundaries. The two Bayesian approaches were also generally well-powered; however, this was accompanied by an inflation the type I error rate.

Under both scenarios, the HP design always resulted in the largest sample size due to a very low chance of making an early decision, whether correct or not. The OBF design was generally more likely to make an early correct decision and hence more likely to reduce the expected sample size compared with the HSD design under the null scenario, however, it became less advantageous under the alternative scenario. The PostP design showed the smallest sample size at most numbers of interims, but compared with the PredP design, it poorly balanced the chance of making an early correct decision and the type I error rate with more than one interim analyses under the null scenario, and it also poorly balanced the chance of making an early correct decision and the type II error rate for the NICE-SUGAR trial under the alternative scenario.

Under the alternative scenario, the estimated treatment effect was found to be increasingly negatively biased from the true effect (i.e. further from an OR of 1 suggesting a stronger treatment effect) as the number of interims increases. This issue with overestimation of treatment effect in adaptive designs is important but less well-studied compared to type I error rate inflation, according to the recent FDA guidance on adaptive designs [33] The bias in estimation of treatment effect can be corrected using various techniques including the median unbiased estimator [34], shrinkage approach [35] and bootstrapping [36]. More recently, Robertson et al. have provided a comprehensive overview of currently available unbiased and bias-reduced estimators of treatment effect for various types of adaptive designs as well as guidance on how to implement and report them in practice [37].

A strength of our study was that it was motivated by two real large-scale high-quality trials representative of late-phase critical care trials. The ADRENAL trial and the NICE-SUGAR trial were used to compare the performance of common group-sequential methods as well as Bayesian approaches used for interim monitoring. Therefore, our findings reflect potential real-life trial application that can also be applied to the design of two-arm trials with other types of outcomes including time-to-event or continuous variables. They are also generalisable to settings outside of critical care.

However, our study has some limitations. First, our simulation study assumed a fixed sample size based on the original ADRENAL and NICE-SUGAR designs. In practice, one would typically increase the target sample size at the design stage to account for the number of interim looks to maintain the desired power. Second, we used a limited number of adaptive methods in our study, focusing on five commonly used frequentist and Bayesian approaches. In addition, approaches such as the HSD design and the Bayesian approaches can be further calibrated by changing parameters or thresholds for stoppings. In particular, the two Bayesian approaches require pre-specification of the thresholds for early stopping. We chose thresholds that have been used in previous studies; however, we found that, as we increased the number of interim analyses the type I and type II error rates were not always preserved.. Third, all interims were assumed to be equally spaced; however, this rarely applies in practice due to non-uniform recruitment rates or logistic considerations. Further work is required to assess whether unequal spacing further decreases the expected sample size. Fourth, our simulations assumed that recruitment was paused at the time of each interim analysis. In practice, most critical care trials would continue recruitment while an interim analysis is performed thus attenuating potential reductions in sample size. Others have discussed this aspect and demonstrated the additional efficiency gains when pausing recruitment at the time of an interim analysis [38, 39]. Wason et al. have also highlighted the logistical complexity of adaptive designs and suggested a careful consideration of efficiency benefits versus potential drawbacks [40]. Fourth, we considered mortality as the sole outcome as is standard for late-phase critical care trials; however, one might also wish to consider the overall cost-effectiveness of adaptive approaches [41]. Flight et al. have recently conducted a simulation study to assess the cost-effectiveness of group-sequential designs at different numbers of interim analyses by constructing an adjusted health economic model [42]. Fifith, important subgroup differences might have been missed due to a loss of statistical power following an early termination of a critical care RCT.

Granholm et. al have recently discussed potential practical drawbacks of critical care trials including oversimplifications of the trials and a lack of harmonisation of trial protocol and methodological drawbacks including overly optimistic effect sizes, limited attention on patient health state outcomes and a lack of flexibility and adaptability. An additional concern, discrepancies between statistical significance and clinical importance caused by dichotomising results using frequentist group-sequential methods has been widely discussed in their research. They also argued that using Bayesian statistical methods result in more nuanced interpretations which avoid dichotomisation, with the use of different priors which enables the formation of context-dependent conclusions as well as different appropriate evidence thresholds depending on the intervention [3].

Ryan et. al recommended a wider application of Bayesian adaptive approaches to phase III critical care trials, based on their case study on the High Frequency OSCillation in Acute Respiratory distress syndrome (OSCAR) trial via simulation [27]. They concluded that Bayesian adaptive designs led to an earlier termination of the trial and a reduced number of patients recruited with over 15% fewer deaths than the original trial design which used two interim analyses with OBF sequential boundaries for early efficacy stopping; the Bayesian adaptive design yielded a similar power and trial conclusions to the original design. Ryan et. al also discussed the impact of the number of interim analyses on the type I error rate inflation using Bayesian adaptive designs without adjustments for multiplicities. They recommended stopping boundary adjustments when using Bayesian adaptive designs that allow for early stopping for efficacy as the number of interims increases in order to control the type I error rate, while no demonstration of the control may be required when using a strict Bayesian approach [43]. Furthermore, Saville et. al demonstrated the advantages of using predictive probabilities compared to group sequential approaches and posterior probability approaches in clinical decision-making process since it appropriately accounts for auxiliary variables and lagged outcomes despite potential computational burdens [26].

Depending on the clinical context and the acceptable risk–benefit balance, it is unlikely that a single approach will consistently outperform all others. In many cases, a group-sequential design with spending functions and an appropriate number of interim analysis, should provide the right balance between correct early stopping and type I or type II error rates. For situations requiring additional flexibility, Bayesian approaches such as PredP and PostP might be more suitable; however, they require more extensive simulations to ensure appropriate calibration of the error rates. Further sensitivity analyses are required for examining the impact of prior distributions including uninformative or informative, positive, neutral or negative, evidence-based or sceptic priors [44].

## Conclusions

In conclusion, this study shows that increasing the number of interim analyses with appropriate efficacy and futility boundaries increases the chance of finding the correct answer at an earlier stage thus decreasing the trial sample size and conserving resources. This is highly applicable to late-phase critical care trials which tend to have large sample sizes and outcomes observed over a relatively short follow-up time.

Systematically exploring adaptive methods when designing critical care trials will aid the choice of the most appropriate method. This should be done in consultation with clinicians in order to identify the design with the most desirable properties and while balancing the benefits due to sample size reductions against potential operational and computational burdens.

### Supplementary Information

**Additional file 1.**

## Data Availability

The data that support the findings of this study are not openly available due to reasons of sensitivity and are available from the corresponding author upon reasonable request. Data are located in controlled access data storage at the George Institute for Global Health.

## Abbreviations

RCTs: Randomised controlled trials
CHESS: HA-1A Efficacy in Septic Shock
ADRENAL: Adjunctive Corticosteriod Treatment in Critically Ill Patients with Septic Shock
NICE-SUGAR: Normoglyceamia in Intensive Care Evaluation-Survival Using Glucose Algorithm Regulation
REMAP-CAP: Randomized, Embedded, Multifactorial Adaptive Platform Trial for Community-Acquired Pneumonia
ASCOT: Australasian Covid-19 Trial
HP: Haybittle-Peto (HP)
OBF: O’Brien-Fleming
HSD: Hwang-Shih-DeCani
PostP: Posterior Probability
PredP: Predictive Probability
OR: Odds ratio
MCMC: Markov Chain Monte Carlo
OSCAR: High Frequency OSCillation in Acute Respiratory distress syndrome
ECD: Early correct decision
EID: Early incorrect decision
