# Supplementary material for: Adaptive designs in critical care trials: a simulation study
Source: BMC Med Res Methodol. 2023 Oct 18;23:236. doi: 10.1186/s12874-023-02049-6 (PMC10585789; doi:10.1186/s12874-023-02049-6)
Supplement: Supplementary file 1 — Additional file 1. [file 12874_2023_2049_MOESM1_ESM.docx]

# Additional file 1

## Type I error rate and Power


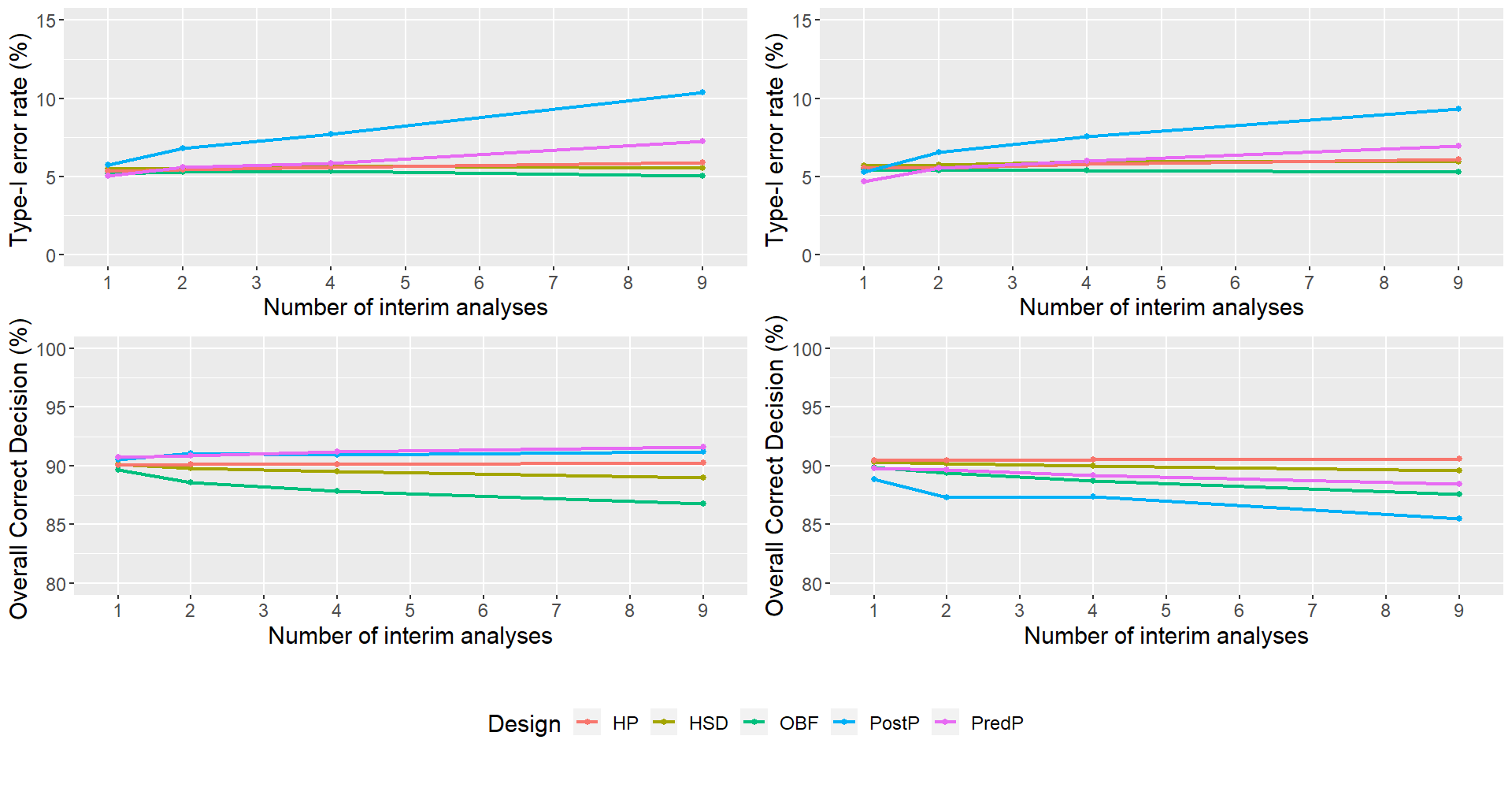


Upper: type I error rate; lower: power; left: ADRENAL; right: NICE-SUGAR.

## Expected sample size (with the reduction from the maximum in %)

ADRENAL (null – total sample size=3658)

|  | **1 interim** | **2 interims** | **4 interims** | **9 interims** |
| --- | --- | --- | --- | --- |
| **OBF** | 3264 (10.8%) | 2827 (22.7%) | 2612 (28.6%) | 2401 (34.4%) |
| **HSD** | 3572 (2.4%) | 3029 (17.2%) | 2850 (22.1%) | 2698 (26.2%) |
| **HP** | 3652 (0.2%) | 3647 (0.3%) | 3641 (0.5%) | 3628 (0.8%) |
| **PostP** | 3120 (14.7%) | 2981 (18.5%) | 2650 (27.6%) | 2364 (35.4%) |
| **PredP** | 3564 (2.6%) | 3419 (6.5%) | 3280 (10.3%) | 3054 (16.5%) |

NICE-SUGAR (null – total sample size=6022)

|  | **1 interim** | **2 interims** | **4 interims** | **9 interims** |
| --- | --- | --- | --- | --- |
| **OBF** | 5356 (11.1%) | 4624 (23.2%) | 4275 (29.0%) | 3936 (34.6%) |
| **HSD** | 5857 (2.7%) | 4995 (17.1%) | 4671 (22.4%) | 4428 (26.5%) |
| **HP** | 6013 (0.1%) | 6010 (0.2%) | 5993 (0.5%) | 5970 (0.9%) |
| **PostP** | 4327 (28.1%) | 3801 (36.9%) | 3476 (42.3%) | 2929 (51.4%) |
| **PredP** | 4936 (18.0%) | 4575 (24.0%) | 4196 (30.3%) | 3692 (38.7%) |

ADRENAL (alternative – total sample size=3658)

|  | **1 interim** | **2 interims** | **4 interims** | **9 interims** |
| --- | --- | --- | --- | --- |
| **OBF** | 3159 (13.6%) | 2869 (21.6%) | 2626 (28.2%) | 2451 (33.0%) |
| **HSD** | 3050 (16.6%) | 2797 (23.5%) | 2596 (29.0%) | 2450 (33.0%) |
| **HP** | 3205 (12.4%) | 3022 (17.4%) | 2844 (22.3%) | 2671 (27.0%) |
| **PostP** | 2700 (26.2%) | 2407 (34.2%) | 2119 (42.1%) | 1828 (50.0%) |
| **PredP** | 3107 (15.1%) | 2894 (20.9%) | 2654 (27.4%) | 2410 (34.1%) |

NICE-SUGAR (alternative – total sample size=6022)

|  | **1 interim** | **2 interims** | **4 interims** | **9 interims** |
| --- | --- | --- | --- | --- |
| **OBF** | 5196 (13.7%) | 4709 (21.8%) | 4316 (28.3%) | 4027 (33.1%) |
| **HSD** | 5014 (16.7%) | 4587 (23.8%) | 4265 (29.2%) | 4021 (33.2%) |
| **HP** | 5278 (12.4%) | 4972 (17.4%) | 4678 (22.3%) | 4381 (27.3%) |
| **PostP** | 4396 (27.0%) | 3775 (37.3%) | 3298 (45.2%) | 2786 (53.7%) |
| **PredP** | 5062 (15.9%) | 4646 (22.8%) | 4245 (29.5%) | 3817 (36.6%) |

## Incremental probabilities of stopping for efficacy or futility at each stage

ADRENAL (null)

| **Number of interims/Analysis stage** | **OBF** | | **HSD** | | **HP** | | **PostP** | | **PredP** | |
| --- | --- | --- | --- | --- | --- | --- | --- | --- | --- | --- |
|  | **P(E)** | **P(F)** | **P(E)** | **P(F)** | **P(E)** | **P(F)** | **P(E)** | **P(F)** | **P(E)** | **P(F)** |
| **1 interim** |  |  |  |  |  |  |  |  |  |  |
| Stage 1 | 0.3 | 21.2 | 0.7 | 4.0 | 0.3 | / | 1.7 | 27.7 | 0.3 | 4.8 |
| Final stage | 4.9 | 73.6 | 4.8 | 90.5 | 5.1 | / | 4.0 | 66.6 | 4.7 | 90.2 |
| **Overall** | **5.2** | **94.8** | **5.5** | **94.5** | **5.4** | **/** | **5.7** | **94.3** | **5.0** | **95.0** |
| **2 interims** |  |  |  |  |  |  |  |  |  |  |
| Stage 1 | 0.0 | 0.0 | 0.3 | 0.0 | 0.3 | / | 2.0 | 4.0 | 0.8 | 0.0 |
| Stage 2 | 1.4 | 66.7 | 1.2 | 49.8 | 0.3 | / | 1.3 | 42.0 | 0.1 | 17.9 |
| Final stage | 3.8 | 28.0 | 4.0 | 44.7 | 4.8 | / | 3.4 | 47.2 | 4.7 | 76.5 |
| **Overall** | **5.3** | **94.7** | **5.5** | **94.5** | **5.4** | **/** | **6.8** | **93.2** | **5.6** | **94.4** |
| **4 interims** |  |  |  |  |  |  |  |  |  |  |
| Stage 1 | 0.0 | 0.0 | 0.1 | 0.0 | 0.2 | / | 1.9 | 0.0 | 1.0 | 0.0 |
| Stage 2 | 0.1 | 0.0 | 0.3 | 0.0 | 0.3 | / | 1.2 | 13.8 | 0.4 | 0.0 |
| Stage 3 | 0.7 | 54.1 | 0.7 | 30.0 | 0.2 | / | 1.0 | 30.1 | 0.1 | 13.0 |
| Stage 4 | 2.0 | 30.9 | 1.5 | 46.3 | 0.2 | / | 0.9 | 21.8 | 0.0 | 20.1 |
| Final stage | 2.5 | 9.7 | 3.1 | 18.0 | 4.7 | / | 2.6 | 26.7 | 4.3 | 61.0 |
| **Overall** | **5.3** | **94.7** | **5.7** | **94.3** | **5.6** | **/** | **7.7** | **92.3** | **5.8** | **94.2** |
| **9 interims** |  |  |  |  |  |  |  |  |  |  |
| Stage 1 | 0.0 | 0.0 | 0.0 | 0.0 | 0.2 | / | 2.1 | 0.0 | 1.5 | 0.0 |
| Stage 2 | 0.0 | 0.0 | 0.0 | 0.0 | 0.2 | / | 1.7 | 0.0 | 0.7 | 0.0 |
| Stage 3 | 0.0 | 0.0 | 0.1 | 0.0 | 0.2 | / | 1.0 | 0.3 | 0.5 | 0.0 |
| Stage 4 | 0.1 | 0.0 | 0.2 | 0.0 | 0.2 | / | 0.8 | 14.1 | 0.2 | 0.0 |
| Stage 5 | 0.2 | 25.2 | 0.2 | 0.0 | 0.1 | / | 0.8 | 19.2 | 0.1 | 4.9 |
| Stage 6 | 0.5 | 30.8 | 0.4 | 29.3 | 0.1 | / | 0.8 | 15.7 | 0.1 | 11.7 |
| Stage 7 | 0.9 | 18.5 | 0.7 | 27.6 | 0.1 | / | 0.6 | 10.9 | 0.1 | 13.4 |
| Stage 8 | 1.1 | 11.3 | 0.9 | 19.9 | 0.1 | / | 0.5 | 7.7 | 0.0 | 10.1 |
| Stage 9 | 1.1 | 6.4 | 1.1 | 12.1 | 0.1 | / | 0.4 | 5.5 | 0.0 | 8.4 |
| Final stage | 1.2 | 2.7 | 1.8 | 5.5 | 4.5 | / | 1.8 | 16.1 | 4.0 | 44.3 |
| **Overall** | **5.1** | **94.9** | **5.5** | **94.5** | **5.9** | **/** | **10.4** | **89.6** | **7.3** | **92.7** |

NICE-SUGAR (null)

| **Number of interims/Analysis stage** | **OBF** | | **HSD** | | **HP** | | **PostP** | | **PredP** | |
| --- | --- | --- | --- | --- | --- | --- | --- | --- | --- | --- |
|  | **P(E)** | **P(F)** | **P(E)** | **P(F)** | **P(E)** | **P(F)** | **P(E)** | **P(F)** | **P(E)** | **P(F)** |
| **1 interim** |  |  |  |  |  |  |  |  |  |  |
| Stage 1 | 0.3 | 21.8 | 0.7 | 4.8 | 0.3 | / | 1.9 | 54.3 | 0.5 | 35.6 |
| Final stage | 5.1 | 72.8 | 5.0 | 89.5 | 5.3 | / | 3.7 | 40.4 | 4.2 | 59.7 |
| **Overall** | **5.4** | **94.6** | **5.7** | **94.3** | **5.5** | **/** | **5.3** | **94.7** | **4.7** | **95.3** |
| **2 interims** |  |  |  |  |  |  |  |  |  |  |
| Stage 1 | 0.0 | 0.0 | 0.2 | 0.0 | 0.2 | / | 1.9 | 30.0 | 0.7 | 14.6 |
| Stage 2 | 1.4 | 68.2 | 1.2 | 49.6 | 0.2 | / | 1.7 | 45.0 | 0.2 | 41.2 |
| Final stage | 4.0 | 26.4 | 4.4 | 44.6 | 5.2 | / | 2.9 | 18.4 | 4.7 | 38.6 |
| **Overall** | **5.4** | **94.6** | **5.7** | **94.3** | **5.5** | **/** | **6.6** | **93.4** | **5.5** | **94.5** |
| **4 interims** |  |  |  |  |  |  |  |  |  |  |
| Stage 1 | 0.0 | 0.0 | 0.1 | 0.0 | 0.4 | / | 2.1 | 1.2 | 1.1 | 0.0 |
| Stage 2 | 0.1 | 0.0 | 0.2 | 0.0 | 0.1 | / | 1.6 | 39.5 | 0.4 | 23.6 |
| Stage 3 | 0.7 | 55.3 | 0.6 | 30.8 | 0.2 | / | 1.0 | 29.1 | 0.2 | 28.6 |
| Stage 4 | 1.9 | 31.0 | 1.4 | 46.8 | 0.2 | / | 0.9 | 13.7 | 0.1 | 17.1 |
| Final stage | 2.8 | 8.3 | 3.6 | 16.5 | 4.9 | / | 2.0 | 8.9 | 4.1 | 24.7 |
| **Overall** | **5.4** | **94.6** | **5.9** | **94.1** | **5.8** | **/** | **7.6** | **92.4** | **6.0** | **94.0** |
| **9 interims** |  |  |  |  |  |  |  |  |  |  |
| Stage 1 | 0.0 | 0.0 | 0.0 | 0.0 | 0.3 | / | 2.0 | 0.0 | 1.4 | 0.0 |
| Stage 2 | 0.0 | 0.0 | 0.1 | 0.0 | 0.3 | / | 1.5 | 1.4 | 1.0 | 0.0 |
| Stage 3 | 0.0 | 0.0 | 0.1 | 0.0 | 0.2 | / | 1.1 | 22.9 | 0.5 | 9.3 |
| Stage 4 | 0.0 | 0.0 | 0.1 | 0.0 | 0.1 | / | 0.8 | 23.4 | 0.3 | 19.3 |
| Stage 5 | 0.2 | 25.3 | 0.2 | 0.0 | 0.2 | / | 0.8 | 15.3 | 0.1 | 17.5 |
| Stage 6 | 0.4 | 31.5 | 0.3 | 29.4 | 0.1 | / | 0.5 | 10.3 | 0.1 | 12.4 |
| Stage 7 | 0.8 | 18.9 | 0.7 | 28.7 | 0.2 | / | 0.6 | 6.6 | 0.0 | 8.2 |
| Stage 8 | 1.0 | 11.2 | 0.8 | 19.8 | 0.1 | / | 0.4 | 4.1 | 0.0 | 6.2 |
| Stage 9 | 1.2 | 5.4 | 1.3 | 11.4 | 0.0 | / | 0.4 | 3.2 | 0.0 | 4.9 |
| Final stage | 1.5 | 2.4 | 2.4 | 4.7 | 4.7 | / | 1.1 | 3.4 | 3.6 | 15.2 |
| **Overall** | **5.3** | **94.7** | **6.0** | **94.0** | **6.1** | **/** | **9.3** | **90.7** | **7.0** | **93.0** |

ADRENAL (alternative)

| **Number of interims/Analysis stage** | **OBF** | | **HSD** | | **HP** | | **PostP** | | **PredP** | |
| --- | --- | --- | --- | --- | --- | --- | --- | --- | --- | --- |
|  | **P(E)** | **P(F)** | **P(E)** | **P(F)** | **P(E)** | **P(F)** | **P(E)** | **P(F)** | **P(E)** | **P(F)** |
| **1 interim** |  |  |  |  |  |  |  |  |  |  |
| Stage 1 | 25.8 | 1.5 | 33.0 | 0.2 | 24.7 | / | 50.6 | 1.8 | 29.9 | 0.2 |
| Final stage | 63.8 | 8.9 | 57.1 | 9.7 | 65.3 | / | 39.9 | 7.7 | 60.8 | 9.1 |
| **Overall** | **89.6** | **10.4** | **90.1** | **9.9** | **90.1** | **/** | **90.5** | **9.5** | **90.7** | **9.3** |
| **2 interims** |  |  |  |  |  |  |  |  |  |  |
| Stage 1 | 3.4 | 0.0 | 13.1 | 0.0 | 13.3 | / | 34.0 | 0.3 | 20.9 | 0.0 |
| Stage 2 | 52.6 | 5.3 | 42.0 | 2.5 | 25.5 | / | 32.3 | 1.6 | 20.3 | 0.4 |
| Final stage | 32.6 | 6.1 | 34.8 | 7.7 | 51.3 | / | 24.7 | 7.1 | 49.6 | 8.7 |
| **Overall** | **88.6** | **11.4** | **89.8** | **10.2** | **90.1** | **/** | **91.1** | **8.9** | **90.9** | **9.1** |
| **4 interims** |  |  |  |  |  |  |  |  |  |  |
| Stage 1 | 0.0 | 0.0 | 3.6 | 0.0 | 6.0 | / | 19.4 | 0.0 | 14.1 | 0.0 |
| Stage 2 | 9.8 | 0.0 | 15.2 | 0.0 | 13.3 | / | 23.8 | 1.4 | 14.8 | 0.0 |
| Stage 3 | 34.8 | 4.0 | 26.4 | 1.5 | 15.9 | / | 20.0 | 1.4 | 12.4 | 0.3 |
| Stage 4 | 29.1 | 4.8 | 26.1 | 3.5 | 15.6 | / | 13.7 | 0.5 | 10.2 | 0.3 |
| Final stage | 14.1 | 3.4 | 18.3 | 5.5 | 39.4 | / | 14.0 | 5.7 | 39.6 | 8.1 |
| **Overall** | **87.9** | **12.1** | **89.5** | **10.5** | **90.1** | **/** | **91.0** | **9.0** | **91.2** | **8.8** |
| **9 interims** |  |  |  |  |  |  |  |  |  |  |
| Stage 1 | 0.0 | 0.0 | 0.7 | 0.0 | 2.1 | / | 10.4 | 0.0 | 8.5 | 0.0 |
| Stage 2 | 0.0 | 0.0 | 2.2 | 0.0 | 4.8 | / | 13.0 | 0.0 | 9.0 | 0.0 |
| Stage 3 | 1.6 | 0.0 | 5.6 | 0.0 | 6.7 | / | 12.7 | 0.0 | 8.2 | 0.0 |
| Stage 4 | 8.0 | 0.0 | 9.1 | 0.0 | 7.7 | / | 12.3 | 1.2 | 7.9 | 0.0 |
| Stage 5 | 15.9 | 1.9 | 12.3 | 0.0 | 8.1 | / | 10.3 | 1.3 | 6.6 | 0.1 |
| Stage 6 | 18.6 | 2.6 | 14.4 | 1.5 | 8.3 | / | 8.5 | 0.8 | 5.6 | 0.3 |
| Stage 7 | 16.2 | 2.5 | 13.7 | 1.5 | 7.8 | / | 6.7 | 0.5 | 5.4 | 0.3 |
| Stage 8 | 12.7 | 2.6 | 12.5 | 2.2 | 7.5 | / | 5.7 | 0.3 | 4.7 | 0.2 |
| Stage 9 | 8.4 | 2.1 | 10.3 | 2.8 | 6.9 | / | 4.6 | 0.2 | 4.0 | 0.1 |
| Final stage | 5.4 | 1.7 | 8.4 | 3.0 | 30.3 | / | 7.2 | 4.5 | 31.7 | 7.4 |
| **Overall** | **86.7** | **13.3** | **89.0** | **11.0** | **90.2** | **/** | **91.3** | **8.7** | **91.6** | **8.4** |

NICE-SUGAR (alternative)

| **Number of interims/Analysis stage** | **OBF** | | **HSD** | | **HP** | | **PostP** | | **PredP** | |
| --- | --- | --- | --- | --- | --- | --- | --- | --- | --- | --- |
|  | **P(E)** | **P(F)** | **P(E)** | **P(F)** | **P(E)** | **P(F)** | **P(E)** | **P(F)** | **P(E)** | **P(F)** |
| **1 interim** |  |  |  |  |  |  |  |  |  |  |
| Stage 1 | 25.8 | 1.6 | 33.1 | 0.4 | 24.7 | / | 48.6 | 5.3 | 29.1 | 2.8 |
| Final stage | 64.0 | 8.5 | 57.3 | 9.3 | 65.8 | / | 40.2 | 5.8 | 60.7 | 7.4 |
| **Overall** | **89.9** | **10.1** | **90.3** | **9.7** | **90.4** | **/** | **88.8** | **11.2** | **89.8** | **10.2** |
| **2 interims** |  |  |  |  |  |  |  |  |  |  |
| Stage 1 | 3.3 | 0.0 | 13.0 | 0.0 | 13.2 | / | 32.8 | 4.8 | 21.4 | 1.8 |
| Stage 2 | 53.8 | 5.0 | 43.0 | 2.5 | 25.9 | / | 33.3 | 3.4 | 20.5 | 1.5 |
| Final stage | 32.3 | 5.7 | 34.2 | 7.3 | 51.4 | / | 21.2 | 4.5 | 47.7 | 7.1 |
| **Overall** | **89.4** | **10.6** | **90.2** | **9.8** | **90.5** | **/** | **87.3** | **12.7** | **89.6** | **10.4** |
| **4 interims** |  |  |  |  |  |  |  |  |  |  |
| Stage 1 | 0.0 | 0.0 | 3.5 | 0.0 | 5.8 | / | 19.7 | 0.1 | 13.9 | 0.0 |
| Stage 2 | 10.0 | 0.0 | 14.8 | 0.0 | 12.9 | / | 23.9 | 5.4 | 15.0 | 2.7 |
| Stage 3 | 34.9 | 3.8 | 26.9 | 1.6 | 16.7 | / | 19.7 | 2.5 | 12.3 | 1.7 |
| Stage 4 | 30.0 | 4.2 | 27.3 | 3.0 | 16.1 | / | 13.1 | 1.4 | 10.4 | 0.6 |
| Final stage | 13.8 | 3.4 | 17.5 | 5.3 | 39.0 | / | 11.0 | 3.2 | 37.7 | 5.8 |
| **Overall** | **88.7** | **11.3** | **90.0** | **10.0** | **90.5** | **/** | **87.4** | **12.6** | **89.2** | **10.8** |
| **9 interims** |  |  |  |  |  |  |  |  |  |  |
| Stage 1 | 0.0 | 0.0 | 0.6 | 0.0 | 2.3 | / | 9.8 | 0.0 | 7.9 | 0.0 |
| Stage 2 | 0.0 | 0.0 | 2.4 | 0.0 | 4.5 | / | 12.7 | 0.2 | 8.8 | 0.0 |
| Stage 3 | 1.6 | 0.0 | 5.5 | 0.0 | 6.9 | / | 13.1 | 4.0 | 8.6 | 1.3 |
| Stage 4 | 8.5 | 0.0 | 8.6 | 0.0 | 7.1 | / | 11.6 | 3.4 | 7.3 | 2.2 |
| Stage 5 | 15.5 | 2.0 | 12.5 | 0.0 | 8.6 | / | 10.5 | 1.8 | 6.6 | 1.3 |
| Stage 6 | 18.4 | 2.2 | 14.1 | 1.5 | 8.4 | / | 8.3 | 1.2 | 6.2 | 0.7 |
| Stage 7 | 17.0 | 2.2 | 14.9 | 1.5 | 8.3 | / | 6.4 | 0.9 | 5.2 | 0.4 |
| Stage 8 | 13.4 | 2.4 | 13.3 | 1.9 | 7.8 | / | 4.8 | 0.7 | 5.0 | 0.2 |
| Stage 9 | 8.5 | 2.1 | 10.1 | 2.8 | 7.2 | / | 3.8 | 0.5 | 4.3 | 0.2 |
| Final stage | 4.9 | 1.6 | 7.6 | 2.8 | 29.5 | / | 4.8 | 1.8 | 28.6 | 5.3 |
| **Overall** | **87.6** | **12.4** | **89.6** | **10.4** | **90.6** | **/** | **85.6** | **14.4** | **88.5** | **11.5** |

P(E) = probability of stopping for efficacy; P(F) probability of stopping for futility; P(F) shows ‘/’ due to the absence of futility boundaries for the Haybittle-Peto design.

## Bias in treatment effect estimates

ADRENAL (null - true OR=1.000)

|  | **1 interim** | **2 interims** | **4 interims** | **9 interims** |
| --- | --- | --- | --- | --- |
| **OBF** | 1.004 | 1.004 | 1.004 | 0.999 |
| **HSD** | 1.004 | 1.003 | 1.004 | 0.998 |
| **HP** | 1.004 | 1.004 | 1.004 | 1.005 |
| **PostP** | 1.003 | 1.002 | 1.002 | 1.008 |
| **PredP** | 1.003 | 1.001 | 1.002 | 1.006 |

NICE-SUGAR (null - true OR=1.000)

|  | **1 interim** | **2 interims** | **4 interims** | **9 interims** |
| --- | --- | --- | --- | --- |
| **OBF** | 1.002 | 1.002 | 1.002 | 0.995 |
| **HSD** | 1.003 | 1.002 | 1.002 | 0.994 |
| **HP** | 1.002 | 1.003 | 1.003 | 1.004 |
| **PostP** | 1.002 | 1.002 | 1.003 | 1.005 |
| **PredP** | 1.002 | 1.002 | 1.002 | 1.004 |

ADRENAL (alternative - true OR=0.790)

|  | **1 interim** | **2 interims** | **4 interims** | **9 interims** |
| --- | --- | --- | --- | --- |
| **OBF** | 0.782 | 0.784 | 0.782 | 0.713 |
| **HSD** | 0.779 | 0.776 | 0.773 | 0.688 |
| **HP** | 0.781 | 0.775 | 0.769 | 0.708 |
| **PostP** | 0.779 | 0.768 | 0.759 | 0.744 |
| **PredP** | 0.779 | 0.771 | 0.761 | 0.745 |

NICE-SUGAR (alternative - true OR=0.830)

|  | **1 interim** | **2 interims** | **4 interims** | **9 interims** |
| --- | --- | --- | --- | --- |
| **OBF** | 0.821 | 0.822 | 0.820 | 0.747 |
| **HSD** | 0.819 | 0.816 | 0.813 | 0.726 |
| **HP** | 0.820 | 0.815 | 0.809 | 0.744 |
| **PostP** | 0.820 | 0.816 | 0.807 | 0.796 |
| **PredP** | 0.820 | 0.815 | 0.807 | 0.795 |
